# Estimating parameters for generalized mass action models with connectivity information

**DOI:** 10.1186/1471-2105-10-140

**Published:** 2009-05-11

**Authors:** Chih-Lung Ko, Eberhard O Voit, Feng-Sheng Wang

**Affiliations:** 1 Department of Chemical Engineering, National Chung Cheng University, Chiayi 621-02, Taiwan; 2 The Wallace H. Coulter Department of Biomedical Engineering at Georgia Institute of Technology and Emory University, 313 Ferst Drive, Atlanta, GA, 30332, USA

## Abstract

**Background:**

Determining the parameters of a mathematical model from quantitative measurements is the main bottleneck of modelling biological systems. Parameter values can be estimated from steady-state data or from dynamic data. The nature of suitable data for these two types of estimation is rather different. For instance, estimations of parameter values in pathway models, such as kinetic orders, rate constants, flux control coefficients or elasticities, from steady-state data are generally based on experiments that measure how a biochemical system responds to small perturbations around the steady state. In contrast, parameter estimation from dynamic data requires time series measurements for all dependent variables. Almost no literature has so far discussed the combined use of both steady-state and transient data for estimating parameter values of biochemical systems.

**Results:**

In this study we introduce a constrained optimization method for estimating parameter values of biochemical pathway models using steady-state information and transient measurements. The constraints are derived from the flux connectivity relationships of the system at the steady state. Two case studies demonstrate the estimation results with and without flux connectivity constraints. The unconstrained optimal estimates from dynamic data may fit the experiments well, but they do not necessarily maintain the connectivity relationships. As a consequence, individual fluxes may be misrepresented, which may cause problems in later extrapolations. By contrast, the constrained estimation accounting for flux connectivity information reduces this misrepresentation and thereby yields improved model parameters.

**Conclusion:**

The method combines transient metabolic profiles and steady-state information and leads to the formulation of an inverse parameter estimation task as a constrained optimization problem. Parameter estimation and model selection are simultaneously carried out on the constrained optimization problem and yield realistic model parameters that are more likely to hold up in extrapolations with the model.

## Background

The ultimate goal of biochemical modeling is the construction of mathematical representations that quantitatively describe the dynamic behaviors of pathway systems. Toward this goal, metabolic reactions are formulated as rate laws, and their kinetic parameters are estimated from experimental data *in vitro *or *in vivo*. Various optimization algorithms, such as gradient-based methods [1], genetic algorithms [2], branch-and-bound methods [3], Newton-flow analysis [4], decomposition approaches [5], multiple shooting methods [6], alternating regression techniques [7], decoupling approaches [8,9], collocation methods [10,11], stochastic optimization [12,13], and many other approaches [14-16] have been applied to determine parameters in biochemical systems models. In all these cases, the optimization approach uses an error criterion that is evaluated against experimental data to assess whether the inferred model is able to describe the dynamic behaviors of the system. If the residual error is small, the model is accepted as a valid representation of the system. Theoretically, the more accurate the model, the smaller the prediction error will be.

Parameter sensitivity analysis is sometimes used as a tool for assessing model accuracy. This type of analysis can be described as the study of behaviors of dynamic systems under small perturbations in system parameters. Specific experiments have been proposed to obtain sensitivity measures of the system in order to validate a model. In this context, Fell [17] reviewed experimental techniques for estimating elasticities and control coefficients that are based on changing enzyme activities while only minimally affecting other system properties (see also [18,19]). The two prominent frameworks for metabolic analysis, Biochemical Systems Theory (BST) and Metabolic Control Analysis (MCA), use sensitivity coefficients in the form of logarithmic derivatives to characterize systemic and local properties [20-23]. The systemic sensitivity coefficients are known as control and response coefficients in MCA and as logarithmic gains in BST, while the local sensitivity coefficients are called elasticity coefficients in MCA and kinetic orders in BST. These coefficients are the basis of two important properties of steady-state metabolic systems, namely the summation and the connectivity relationships, which were discovered in MCA and similarly hold in BST for the majority of practical examples (see [24] for exceptions). They are intrinsic features of metabolic systems in which the enzymes affect reactions in a linear fashion. The summation relationship is a local property that states that the sum of all sensitivities of a particular flux with respect to all rate constants is always equal to one. A particularly useful and important feature of the connectivity relationship is that it relates the kinetic properties of the individual reactions (local properties) to (global) properties of the intact pathway.

In this study, connectivity information of the pathway system, which is assumed to have been obtained in separate steady-state experiments, is employed as a constraint for improved parameter estimation from dynamic data. This use of experimental connectivity information as a set of *a priori *constraints renders the proposed method distinctly different from a recent method for lin-log models, which uses connectivity information for *a posteriori *tests of the combined dynamic and steady-state parameter estimation [25]. One might surmise that the connectivity constraints could simply be computed for each power-law model during the iterative estimation process. While this is true, the result would not be informative, because the summation and connectivity relationships are "automatically" satisfied if both the flux control coefficients and the elasticity coefficients are obtained from a power-law model [21,22]. The proof is shown in Additional file 1. The key here is that the control coefficients are obtained from independent steady-state experiments and serve as truly additional constraints, thereby augmenting the top-down estimation from time series data with bottom-up information from steady-state data.

## Results and discussion

To examine the effectiveness of the proposed method, we applied the constrained estimation approach to two case studies.

### Case I: Linear pathway

**Figure 1:**
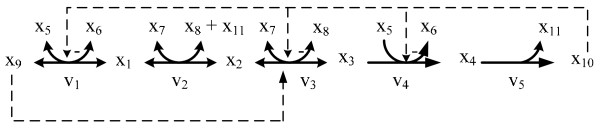
**Linear pathway**. Metabolic reaction steps of the threonine pathway from aspartate in *Escherichia coli*. The dependent variables, *x*_1_, *x*_2_, *x*_3 _and *x*_4_, respectively denote the concentrations of aspartyl-P, D, L-aspartic *β*-semialdehyde, homoserine and O-phospho-homoserine. The independent variables, *x*_5_, *x*_6_, *x*_7_, *x*_8_, *x*_9 _and *x*_10 _represent ATP, ADP, NADPH, NADP, aspartate, threonine and Pi, respectively. (The model is called chassagnole2 in JWS web site).

In the first case study, we determined the rate constants and kinetic orders of the linear steps of the threonine pathway from aspartate in *Escherichia coli*. Threonine is an essential amino acid for birds and mammals, and there is considerable interest in its economic industrial production for a variety of uses. The five-step metabolic pathway for its synthesis from aspartate, as shown in Figure 1, has been studied extensively [26-29]. Each kinetic step in the threonine pathway was originally formulated as a Michaelis-Menten-like model, which is available on the website . From this website, six sets of time-series data were generated using different values for the independent variables. Furthermore, 5% random noise was added to each set of time-series data in order to emulate *in vivo *observations. We reformulated the pathway as the following S-system

(1)

where the dependent variables, *x*_1_, *x*_2_, *x*_3 _and *x*_4_, respectively denote the concentrations of aspartyl-P, D, L-aspartic *β*-semialdehyde, homoserine and O-phospho-homoserine. The independent variables, *x*_5_, *x*_6_, *x*_7_, *x*_8_, *x*_9 _and *x*_10_, represent ATP, ADP, NADPH, NADP, aspartate, threonine and inorganic phosphate (Pi), respectively. The first, second and third rate equations are reversible so they are aggregated into a single term to represent influx/efflux. Using the flux connectivity relationships, as shown in the *Methods *section, we have three additional equality constraints, namely:

(2)

For true values of *S*(*v*_*j*_, *α*_*i*_) and of the kinetic orders *g*_*ij*_, the three *ρ *should be equal to 0 (see Eq. 9). However, if the measured *S*(*v*_*j*_, *α*_*i*_) are combined with estimated *g*_*ij*_, this is not necessarily the case.

For the linear pathway, five fluxes are identical to *v *at steady-state. Twenty-five parameters are included in the constrained parameter estimation problem, which we determine by means of hybrid differential evolution (HDE) [30]. The search range for each rate constant is set to be [0, 5]. The kinetic orders are chosen from with [0, 2], except for *g*_1,10_, *g*_3,10 _and *g*_4,10_, which represent inhibition of the reaction steps *v*_1_, *v*_3 _and *v*_4 _and are therefore negative.

Two computational approaches are applied to determine the 25 parameters. In the first computation, we do not consider the flux connectivity constraints (2), so as to demonstrate a common parameter estimation approach. In the second computation, we assume that the flux control coefficients have been obtained in independent steady-state experiments, as it is typical in MCA [17,31,32]. These experimental control coefficients are now provided as additional information so that the kinetic orders in the parameter estimation problem are restricted by the flux connectivity relationships in (2).

**Figure 2:**
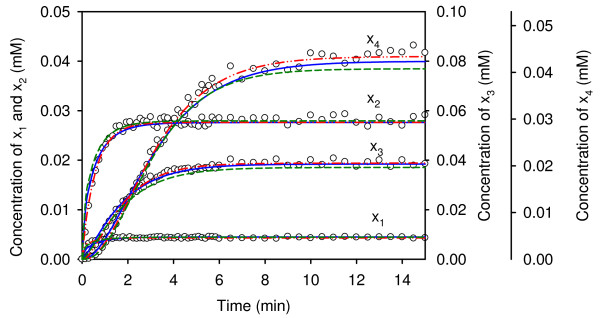
**Model validation for case I**. Model validation using optimal estimates obtained with different computational approaches for an experiment with altered independent variables. Dashed curves represent the predicted profiles using the optimal estimates obtained from estimation without including the flux connectivity constraints. Solid curves represent the predicted profiles using the optimal estimates obtained from an estimation accounting for noise-free flux connectivity constraints. Dashed-dot-dot curves represent the predicted profiles using the optimal estimates obtained from an estimation accounting for 5% noise in the measured flux connectivity constraints. Data points are *in silico *observations. The independent variables [1.376, 0.179, 0.588, 0.630, 1.340, 3.490, 4.7500] were set 5% outside the training range.

For this unconstrained HDE approach, the value of the least-square error criterion was 2.52E-3. The optimal HDE estimates were then provided as the starting point for a gradient-based method to yield a refined solution, which is listed in the first column of Table 1. The least-squared error value was 2.15E-4 for the refined search. In order to validate the fitness of the optimal estimates, additional time-series data were generated. Specifically, the independent variables for the validation test experiment were set 5% outside the training ranges. Figure 2 shows the predictive dynamic profiles (dashed curves) and the *in silico *experimental data. The least-square error was 5.57E-3 for the validation. The optimal estimates were also applied to compute each constraint *ρ *(Eqs. 2) with the experimental flux connectivity coefficients. As shown in Table 2, to the estimated values of *ρ *yielded a sum of the constraint violations (SCV) of 4.28E-1 (see *Methods*). This relatively high SCV value indicates that the flux connectivity constraints are unduly violated.

**Table 1:** Estimated results for Case I

Parameter	Estimation without constraints	Estimation with constraints (noise-free)	Estimation with constraints (5% noise)
*α*_1_	4.67E-05	2.42E-04	3.43E-6
*α*_2_	3.452	0.079	0.436
*α*_3_	3.359	0.733	2.227
*α*_4_	1.828	3.660	1.961
*α*_5_	0.359	0.434	4.955
*g*_1,1_	-0.772	-0.763	-1.437
*g*_1,5_	1.710	0.597	1.896
*g*_1,6_	-1.110	-0.113	-0.665
*g*_1,9_	1.241	0.927	0.868
*g*_1,10_	-0.328	-0.005	-0.757
*g*_2,1_	0.948	0.857	1.59
*g*_2,2_	-0.467	-1.308	-1.996
*g*_2,7_	0.963	0.783	0.407
*g*_2,8_	-1.002	-0.972	-1.26
*g*_2,11_	-0.682	-0.712	-1.235
*g*_3,2_	1.121	0.702	0.987
*g*_3,3_	-0.002	-0.008	-0.012
*g*_3,7_	0.263	0.171	0.515
*g*_3,8_	-0.044	-0.153	-0.850
*g*_3,9_	-0.185	0.106	-0.384
*g*_3,10_	-0.307	-0.607	-0.835
*g*_4,3_	0.871	0.900	1.118
*g*_4,5_	-0.347	-0.586	0.754
*g*_4,10_	-0.819	-1.382	-1.524
*g*_5,4_	0.745	0.859	1.392

**Table 2:** The computed results of the flux connectivity constraints for Case I

Without flux connectivity constraints			With flux connectivity constraints	
No.	noise-free	5% noise	noise-free	5% noise

1	2.016E-2	2.347E-2	-1.242E-4	1.652E-4
2	4.048E-1	4.093E-1	1.117E-4	-3.088E-2
3	3.454E-3	3.625E-3	5.752E-4	3.100E-4

"Noise-free" indicates that the measured flux control coefficients are assumed to be perfect. For "5% noise" it is assumed that the measured flux control coefficients have 5% uncertainty.

The second computation minimized the combined penalty function (10) (see *Methods*), which includes the flux connectivity constraints (2) in the objective function. The HDE algorithm in this case yielded a least-squared error value of 8.63E-3. The refined optimal estimates obtained by the subsequent gradient-based method are listed in the second column of Table 1 and were also applied to evaluate the additional test experiment. The predictive profiles are shown as solid curves of Figure 2. The least-square error was 2.13E-2 for the validation. The flux connectivity constraints are shown in Table 2. Each flux connectivity constraint is smaller than the result obtained from the first computation. A SCV of 8.11E-4 was obtained for this case. This result implies that the parameter values estimated by the constrained optimization approach are more feasible than the first computed results.

The flux control coefficients were assumed to be perfectly measured for the above computation. To emulate *in vivo *observations, 5% random variation was added to the true flux control coefficients. Following the same procedure, the least-square value of 2.57E-2 was obtained by using the imperfect flux control coefficients. The optimal estimates, as shown in Table 1, were also applied to evaluate the additional test experiment with noise. The predicted profiles (dashed-dot-dot curves) are nearly identical to the noise-free results. The flux connectivity constraints are shown in Table 2. Each flux connectivity constraint is smaller than the result obtained from the first computation. A SCV of 3.14E-2 was obtained for this case. However, a SCV of 4.36E-1 was obtained with the unconstrained parameter estimation technique.

### Case II: Branched pathway with feedback

**Figure 3:**
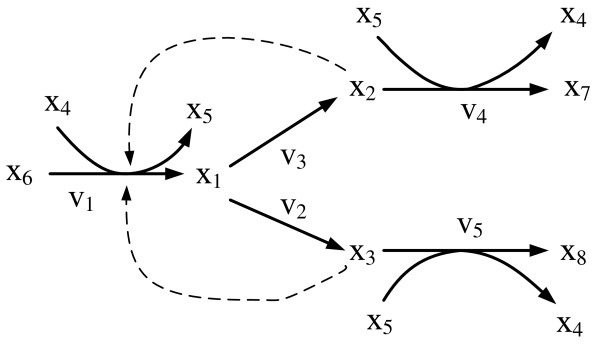
**Branched pathway with feedback**. Metabolic map for branched pathway with five enzymes and feedback. (The model is called feedbackmoi in JWS web site.) *x*_1_,..., *x*_5 _are dependent variables, *x*_6_, *x*_7 _and *x*_8 _are independent variables. Each product is fed back to regulate its corresponding pathway. Both *x*_1 _and *x*_2 _are also fed back to regulate the first reaction pathway.

The second case study consisted of a five-enzyme branched pathway with feedback regulation, as shown in Figure 3. Two feedback signals inhibit the first reaction. Each rate equation was originally formulated as a Michaelis-Menten-like model. From the web site, six sets of time-series data were generated, and 5% random noise was added to each set of time-series data in order to emulate *in vivo *observations. The material balance equations were modeled as

(3)

Each rate equation was formulated for our analysis as a power-law model of the following type:

(4)

There are fifteen flux connectivity relationships for this branched pathway. Following procedures analogous to those discussed in the previous example, the 23 parameters were estimated with both computational approaches; the results are shown in Table 3. The time-series data were generated from the *in silico *system with six sets of initial conditions and independent variables. Both approaches are able to estimate the parameters for the dependent variables and also to determine the kinetic orders related to dependent and independent variables.

**Table 3:** Estimated results for Case II

Parameter	Estimation without constraints	Estimation with constraints (noise-free)	Estimation with constraints (5% noise)
*α*_1_	0.6294	2.0096	5.0
*α*_2_	0.3618	1.0826	1.3442
*α*_3_	0.2680	0.9776	3.9705
*α*_4_	0.0654	0.7542	3.7875
*α*_5_	0.1327	0.9763	1.3159
*g*_11_	1.5285	0.0987	0.0226
*g*_12_	-2.0000	-0.4603	-0.0362
*g*_13_	0.9963	0.1534	0.0635
*g*_14_	0.5945	0.3144	0.0332
*g*_15_	-2.0	-0.1296	-0.0483
*g*_16_	0.0916	0.0396	0.0268
*g*_21_	1.8490	0.1834	0.0458
*g*_23_	0.2849	-0.0720	-0.0299
*g*_31_	1.6939	0.2020	0.0481
*g*_32_	-0.4312	-0.1026	-8.5364E-3
*g*_42_	-1.2477	0.1276	9.456E-3
*g*_44_	1.1855	-0.1892	-0.0209
*g*_45_	0.7681	0.0778	0.0304
*g*_47_	-0.4538	-0.0541	-0.0149
*g*_53_	2.0	0.1082	0.0462
*g*_54_	-1.4214	-0.2074	-0.0275
*g*_55_	1.6615	0.0847	0.0401
*g*_58_	-0.8134	-0.0669	-0.0260

**Figure 4:**
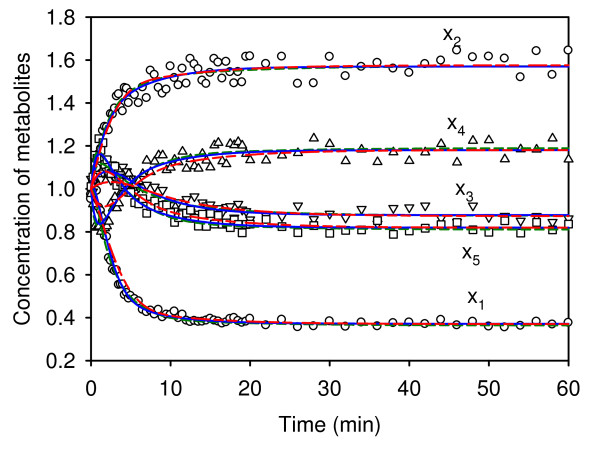
**Model validation for case II**. Model validation using optimal estimates obtained with different computational approaches. Dashed curves represent the predicted profiles using optimal estimates obtained from estimation without accounting for flux connectivity constraints. Solid curves represent the predicted profiles using the optimal estimates obtained from an estimation accounting for noise-free flux connectivity constraints. Dashed-dot-dot curves represent the predicted profiles using the optimal estimates obtained from an estimation accounting for 5% noise in the measured flux connectivity constraints. Data points are *in silico *observations. The independent variables are set 5% outside the training range.

The first computational approach did not consider the flux connectivity constraints. After the global-local search, we obtained a least-squared error of 7.63E-4. The optimal estimates were then applied to compute each constraint in Eq. (9) with the true flux connectivity coefficients, as shown in Table 4; they correspond to an SCV of 14.71, which indicates severe constraint violation. The second computational approach was then applied to solve the parameter estimation problem with minimization of flux connectivity constraints. The least-square error was 1.87E-4, which is smaller than that obtained from the first computation. The 15 constraints in this case are also shown in Table 4. The SCV of 1.41E-3 is much smaller than that of the first method. This means that the optimal estimates from the second approach are more adequate than those obtained from the estimation without considering the flux connectivity constraints. Additional sets of initial conditions and independent variables were used to generate the time-series data to validate both optimal estimates. Figure 4 shows the predicted dynamic profiles (solid and dashed curves) and the *in silico *experimental data. The predicted profiles are nearly identical, even though the estimated parameters are quite different, indicating residual "sloppiness" of the model [33].

**Table 4:** The computed results of the flux connectivity constraints for Case II

Without flux connectivity constraints			With flux connectivity constraints	
No.	noise-free	5% noise	noise-free	5% noise

1	4.735E-1	5.392E-1	5.499E-5	3.573E-3
2	-1.742	-1.785	-3.055E-5	-8.999E-4
3	1.231E-1	1.375E-1	6.428E-5	-2.474E-4
4	1.720	1.745	-2.078E-6	1.351E-3
5	-4.973E-1	-5.237E-1	1.404E-5	-1.694E-3
6	5.514E-1	4.366E-1	1.852E-4	-2.283E-3
7	-1.394	-1.336	-4.564E-5	6.405E-4
8	5.378E-1	5.177E-1	-2.719E-5	1.319E-4
9	1.241	1.209	1.303E-4	-8.199E-4
10	-3.540E-1	-3.401E-1	-1.360E-4	1.042E-3
11	3.681E-1	3.208E-1	-1.211E-4	-1.929E-3
12	-2.213	-2.267	-9.843E-6	3.888E-4
13	-4.378E-1	-4.162E-1	1.878E-4	-6.744E-5
14	2.367	2.411	-1.812E-4	-4.822E-4
15	-6.911E-1	-6.132E-1	2.170E-4	5.719E-4

"Noise-free" indicates that the measured flux control coefficients are perfect, while "5% noise" is based on measured flux control coefficients with 5% uncertainty.

As in the previous case, 5% random variation was added to the perfect flux control coefficients to emulate *in vivo *observations. A least-square value of 1.62E-2 was obtained by using these imperfect flux control coefficients. The optimal estimates, as shown in Table 3, were applied to evaluate the additional test experiment. The predicted profiles (dashed-dot-dot curves) are almost identical to the noise-free results. The flux connectivity constraints are shown in Table 4. Each flux connectivity constraint is smaller than the result obtained from the first computation. A SCV of 1.61E-2 was obtained for this case. However, a SCV of 14.6 was obtained with the common parameter estimation technique. Thus, values obtained through parameter estimation with connectivity constraints can be expected to be more realistic than those obtained by unconstrained techniques.

It is a difficult task to assess the quality of a model if the model predictions for all tested data are good, as discussed in the case studies. If it is not feasible to retain some of the original data for cross-validation, other types of data must be used. For instance, steady-state related experimental information, such as flux control coefficients, may be applied to validate the model through testing connectivity relationship [25]. In our case, these cannot be used for validation, since we are using them as constraints. This leaves additional time-series experiments as the best feasible alternative for mode validation, as we showed above.

## Conclusion

Parameter estimation for biological models is the bridge connecting the wet and the dry labs. There are still many challenges in using time-series data to solve parameter estimation problems for nonlinear biological systems. In many cases, this estimation is a cyclic task. Rate constants and kinetic orders of the differential equations are estimated from time-series data. Some new experiments should then be carried out to validate the estimated model. Inconsistencies may lead to improved second-round estimates, which are again to be validated.

A common problem in parameter estimation is the observation that distinctly different solutions may lead to model fits with very similar residual errors [34]. One reason for this situation is the compensation of error among the terms and/or equations of the model [35]. The problem with different solutions becomes most apparent in extrapolations to new conditions, were misestimated models fail. A complex question is thus which of the estimated models is best. While a general answer cannot be given, our results here show that the prudent consideration of constraints limits the variety of different models with similar residual fit.

Specifically, we use relationships between parameters and parameter sensitivities, which were developed in the modelling framework of MCA. While it is widely known that parameter sensitivity analysis may be used to investigate which parameter in the system is most sensitive, we have shown here that sensitivity analysis may also serve as a valuable set of upfront constraints for improved parameter estimation strategies. We showed with two representative case studies that the consideration of flux connectivity relationships can help constrain the parameter estimation problem and lead to significantly improved model parameters. While we did not use true experimental data for the illustration of the method, we created artificial data from Michaelis-Menten type pathway model representations that were different from the BST models we used for the estimation analyses. Furthermore, we allowed for experimental error, so that the resulting artificial data were as different from the BST models as is reasonably possible in a purely computational setting. The result showed that the consideration of connectivity constraints was computationally cheap and, yet, greatly improved the estimated solutions.

## Methods

### Model formulation

The dynamics of a biochemical reaction system can be represented generically using a set of nonlinear ordinary differential equations with the following structure:

(5)

Here, *x*_*i *_is the metabolite concentration for the *i*^th ^component or pool, *n*_*ij *_are the stoichiometric coefficients and *v*_*j *_is the reaction rate for the *j*^th ^pathway. For most traditional formulations, such rates are represented as constant flux rates, mass action functions or Michaelis-Menten like models. As an alternative, BST employs power-law models to express each rate. BST formulations can thus be represented as:

(6)

where *α*_j _is the rate constant for the *j*^th ^rate-law equation, and *g*_*jk *_are kinetic orders. Yet a different alternative is the use of lin-log models, which are extensions of MCA [25].

Both rate constants and kinetic orders in Eq. (6) can, in general, be estimated from steady-state data or from dynamical data. The nature of suitable data for these two types of estimations is rather different, and so are the methods of analysis. Estimations of parameter values from steady-state data are generally based on experiments that measure how biochemical system responds to small perturbations around the steady state (as a very detailed example, see [36]). Two approaches may be taken. The first tries to measure directly how the variable *x*_*k *_affects the influx into or efflux out of the pool *x*_*j*_, (this effect, by definition, is represented by the kinetic order parameters or elasticities). The second measures logarithmic gains, which describe the influence of an independent variable on a dependent variable. Because gains are closely related to kinetic orders, they provide an indirect measure of relevant system properties as well as the topological structure of the model [37,38]. Estimation from dynamic data is based on quite a different type of experimentation. In this case, time-series measurements are needed for all metabolites of the system [39]. The temporal data may stem from transient responses after a perturbation from steady state [40], but they are more often found in the analysis of systems that exhibit growth, decay, or some other long-term dynamics (e.g., [41]). In this study, we combine both dynamic and steady-state approaches in order to obtain more exact models.

### Error function

Time series based parameter estimation is used to determine rate constants and kinetic orders so that the dynamic profiles satisfactorily fit the measured observations. This task is formulated as an optimization problem to minimize an objective function that measures the goodness of fit of the model with respect to a given experimental time-series dataset. The sum of least-squared errors criterion is a commonly used as the objective function, which is expressed as

(7)

where  is the measurement of the *i*^th ^component at *t *= *t*_*s*_, *x*_*i*_(*t*_*s*_) is the computed concentration for the *i*^th ^component at *t *= *t*_*s*_, and  is the maximum measured concentration of the *i*^th ^component, which is used for normalization purposes so that variables with different scales have a similar impact. *N*_*s *_is the number of sampled data points. The dynamic profiles *x*_*i*_(*t*_*s*_) are typically obtained by applying a numerical integration method to solve the differential equations (5).

### Connectivity constraints

In this study, the power-law model in (6) is employed to formulate each process in a biochemical system. Parameter estimation is then carried out with time-series data to determine the rate constants and kinetic orders of the model that give the best fit to a set of experimental data. The Fisher information matrix is a popular measure for model validation. An alternative approach is parameter sensitivity analysis, which permits the determination of which parameters in the system are most sensitive. Such a parameter sensitivity analysis can be used to validate the model's response and to design experiments that support the estimation of parameters [25].

Within the context of sensitivity analysis, the hallmark results of MCA are the summation and connectivity relationships [42-44]. While they are not applicable in all situations [24], they hold for most typical pathway systems and in these cases can also be derived directly from the mathematical structure of systems formulated within BST [21-23]. The summation and connectivity relationships have been used to provide valuable insights into the behavior of metabolic pathways. Mathematically, they amount to descriptions of sensitivity invariants, and they are consequences of the stoichiometric nature of the system. For a typical pathway system at steady-state, the summation property indicates that the sum of all sensitivities of a particular flux with respect to all rate constants is equal to one:

(8)

The local properties are thus expressed as relationships between all rate constants (which are proportional to enzyme activities) and an individual reaction.

Flux connectivity relationships address global properties. They are expressed as

(9)

where  and  are respectively described in MCA as flux control coefficients and elasticity coefficients. As we demonstrate in this study, these flux connectivity relationships can be used as constraints to assist in the estimation of more accurate and realizable model parameters. Many approaches have been proposed for measuring flux control coefficients (*e.g*., [17,31,32]). Such measurements along with the estimated kinetic orders obtained from parameter estimation can be applied to compute the connectivity relationships to validate the model.

### Optimization with constraints

Many methods could be used as the optimization engine for estimating parameters [39]. Here we decided to use a fast hybrid differential evolution (HDE) for the evaluation of the differential equations, followed by a refining gradient method. The HDE algorithm [30] is a simple, population-based, stochastic method and has been extended from the original algorithm of differential evolution (DE) as described by Storn and Price [45]. The basic operations of DE are similar to conventional evolutionary algorithms. However, HDE includes two additional operations, namely acceleration and migration. Both operations serve as trade-off operators for balancing convergent rate and population diversity in the evolutionary computation. Acceleration is used to speed up the convergent rate. However, faster descent usually results in yielding a premature solution. Thus, migration is used to increase the population diversity to prevent the algorithm from reaching a premature solution. Accordingly, HDE enables a smaller population to be used for finding a global solution and has succeeded in solving several biochemical optimization problems. Details of the HDE algorithm are provided in Additional file 2.

Most nonlinear regressions are performed with gradient-based optimization methods so that the solution quality strongly depends on the provided initial starting point. Moreover, gradient-based methods may yield a local minimum, rather than a global solution. Evolutionary algorithms can be applied to overcome such drawbacks. However, numerical integration failure is the major problem during the evolutionary search progress. In addition, numerical integration is time-consuming. Slope approximation [8,46], decomposition strategies [47] and modified collocation methods [10] can be applied to alleviate the computation burden. Indeed, HDE with modified collocation method has been shown to achieve global estimates quickly. While the solution obtained by a modified collocation method may not always be smooth due to measurement noise, a global-local optimization technique may be applied to overcome this drawback. Tsai and Wang [10] introduced HDE as a global search method to determine a coarse solution and then used the optimal HDE estimates as the starting point for a gradient-based optimization method that employed numerical integration to obtain a refined solution.

In this study, the HDE algorithm is applied to minimize the least error criterion (7) subject to the connectivity constraints (9). As a result, the parameter estimation problem becomes a multivariable minimization problem with equality constraints. Penalty function methods are some of the most popular techniques for handling constraints. The penalty function is defined here as

(10)

where *λ*_*jk *_are positive penalty parameters, and the parameters *θ *denote all rate constants *α*_*i*_, and kinetic orders *g*_*ik *_that are to be determined. In this constrained parameter estimation, we use the sum of the constraint violations (SCV) to inspect the feasibility of the estimated optimal solution. SCV is defined as

(11)

Thus, SCV can be seen as a global measure of the accuracy of connectivity in the model. If all *S*(*v*_*j*_, *α*_i_) and *g*_*ik *_were taken from a given pathway model, SCV would be zero. However, if the *S*(*v*_*j*_, *α*_i_) are obtained from independent steady-state experiments, this is not necessarily the case, and SCV can be used as a constraint that should be as close to zero as possible.

### Computational settings

All computations were carried out on a Pentium IV computer using Microsoft Windows XP. The HDE algorithm was implemented in Compaq Visual Fortran. Four settings require provisions for HDE and are given as follows: the crossover factor was set to 0.5; two tolerances used in the migration were set to 0.05; and a population size of 5 was used in the computation. These settings served as the default values for all computations in the case studies. In HDE, the mutation factor is taken as a random number within [0, 1]. In order to yield smoother profiles, optimal estimates obtained by HDE are then provided as the initial starting point for the gradient-based method, a subroutine BCONF in the IMSL Math/Library, to solve the parameter estimation problem. The gradient-based method employs Runge-Kutta techniques of various orders, along with the subroutine IVMRK in IMSL Math/Library, to solve differential equations towards obtaining time-series profiles of the system.

## Authors' contributions

CLK implemented the algorithm and performed the simulations. EOV provided critical insight into the constrained estimation problem and interpretation of results. FSW proposed the main idea and supervised the numerical tests. All authors contributed to preparation of the manuscript.

## Supplementary Material

Additional file 1 **Flux connectivity relationships**. To show that the flux connectivity relationships are naturally satisfied if both flux control coefficients and elasticity coefficients are obtained from the same GMA model. Click here for file

Additional file 2 **Hybrid differential evolution**. To describes the computational algorithm and the property of convergence for the HDE algorithm. Click here for file
