# Supplementary material for: Estimating parameters for generalized mass action models with connectivity information
Source: BMC Bioinformatics. 2009 May 11;10:140. doi: 10.1186/1471-2105-10-140 (PMC2694188; doi:10.1186/1471-2105-10-140)
Supplement: Additional file 1 — Flux connectivity relationships. To show that the flux connectivity relationships are naturally satisfied if both flux control coefficients and elasticity coefficients are obtained from the same GMA model. [file 1471-2105-10-140-S1.pdf]

# Supplement 1: Flux connectivity relationships

**Estimating Parameters for Generalized Mass Action Models with Connectivity Information**

Chih-Lung Ko, Eberhard O. Voit and Feng-Sheng Wang

The flux connectivity relationships in (S1-1) are naturally satisfied if both flux control coefficients and elasticity coefficients are obtained from the same GMA model.

$$\sum_{i=1}^{r} C_{e_i}^{v_j} \varepsilon_{X_j}^{v_i} = \sum_{i=1}^{r} S\left(v_j, \alpha_i\right) g_{ik} = 0,\ j = 1,\ldots,r; k = 1,\ldots,n \qquad \text{(S1-1)}$$

Any GMA model of a metabolic pathway can be formulated as $\dot{\vec{\mathbf{X}}} = \mathbf{N}\vec{\mathbf{V}}$, where $\dot{\vec{\mathbf{X}}}$ is the vector of derivatives $\dot{X}_i$, $\mathbf{N}$ is a stoichiometric matrix, and $\vec{\mathbf{V}}$ is a vector containing the fluxes $v_j$, each of which has the form $v_j = \alpha_j \prod_{k=1}^{n+m} x_k^{g_{jk}}$ , where $\alpha_j$ are rate constants, and $g_{kj}$ are kinetic orders. Each rate equation can be expressed as $v_j = v_j\left(\vec{\mathbf{X}}_D(\vec{\mathbf{X}}_I, \vec{\mathbf{A}}, \mathbf{F}_D, \mathbf{F}_I)\right)$, where the notations are defined as follows:

$\vec{\mathbf{X}}_D$ : vector of dependent variables

$\vec{\mathbf{X}}_I$ : vector of independent variables

$\vec{\mathbf{A}}$ : vector of rate constants

$\mathbf{F}_D$ : matrix of kinetic orders associated with dependent variables

$\mathbf{F}_I$ : matrix of kinetic orders associated with independent variables

The vectors, $\vec{\mathbf{V}}$, $\vec{\mathbf{X}}$, $\vec{\mathbf{X}}_D$, $\vec{\mathbf{X}}_I$ and $\vec{\mathbf{A}}$ can be expressed in the form of square matrices, $\mathbf{V}$, $\mathbf{X}$, $\mathbf{X}_D$, $\mathbf{X}_I$ and $\mathbf{A}$ by putting their elements in diagonals and setting all other matrix elements equal to zero.

The kinetic matrices $\mathbf{F}_D$ and $\mathbf{F}_I$ can be written as logarithmic derivatives of the individual fluxes with respect to dependent or independent variables. The matrices of elasticity coefficients are therefore expressed as

$$\mathbf{F}_D = \mathbf{V}^{-1}\frac{\partial \vec{\mathbf{V}}}{\partial \vec{\mathbf{X}}_D}\mathbf{X}_D \text{ and } \mathbf{F}_I = \mathbf{V}^{-1}\frac{\partial \vec{\mathbf{V}}}{\partial \vec{\mathbf{X}}_I}\mathbf{X}_I$$

They are equivalent to the following formulation:

$$\mathbf{F}_D = \begin{bmatrix} f_{11} & f_{12} & \cdots & f_{1n} \\ f_{21} & f_{22} & \cdots & f_{2n} \\ \vdots & \vdots & \ddots & \vdots \\ f_{n1} & f_{z2} & \cdots & f_{nn} \end{bmatrix} = \begin{bmatrix} v_1^{-1} & 0 & \cdots & 0 \\ 0 & v_2^{-1} & \ddots & \vdots \\ \vdots & \ddots & \ddots & 0 \\ 0 & \cdots & 0 & v_r^{-1} \end{bmatrix} \begin{bmatrix} \frac{\partial v_1}{\partial X_1} & \frac{\partial v_1}{\partial X_2} & \cdots & \frac{\partial v_1}{\partial X_n} \\ \frac{\partial v_2}{\partial X_1} & \frac{\partial v_2}{\partial X_2} & \cdots & \frac{\partial v_2}{\partial X_n} \\ \vdots & \vdots & \ddots & \vdots \\ \frac{\partial v_r}{\partial X_1} & \frac{\partial v_r}{\partial X_2} & \cdots & \frac{\partial v_r}{\partial X_n} \end{bmatrix} \begin{bmatrix} X_1 & 0 & \cdots & 0 \\ 0 & X_2 & \ddots & \vdots \\ \vdots & \ddots & \ddots & 0 \\ 0 & \cdots & 0 & X_n \end{bmatrix}$$

Sensitivities of concentrations with respect to rate constants can be expressed in matrix form as follows:

Take the derivative of the GMA model with respect to the rate constant vector:

$$\frac{d\dot{\vec{\mathbf{X}}}}{d\vec{\mathbf{A}}} = \frac{d}{dt}\left(\frac{d\vec{\mathbf{X}}}{d\vec{\mathbf{A}}}\right) = \mathbf{N}\left(\frac{\partial\vec{\mathbf{V}}}{\partial\vec{\mathbf{X}}_D}\frac{d\vec{\mathbf{X}}_D}{d\vec{\mathbf{A}}} + \frac{\partial\vec{\mathbf{V}}}{\partial\vec{\mathbf{A}}}\right)$$

Applying normalization, we obtain the sensitivity matrix of the concentrations at steady state as

$$\mathbf{NVV}^{-1}\frac{\partial\vec{\mathbf{V}}}{\partial\vec{\mathbf{X}}_D}\mathbf{X}_D\mathbf{X}_D^{-1}\frac{d\vec{\mathbf{X}}_D}{d\vec{\mathbf{A}}}\mathbf{A} + \mathbf{NVV}^{-1}\frac{\partial\vec{\mathbf{V}}}{\partial\vec{\mathbf{A}}}\mathbf{A} = \mathbf{0}$$

Using definitions for the elasticity matrix and the sensitivity matrix of concentrations, we have

$$\mathbf{S}\left(\vec{\mathbf{X}}_D,\vec{\mathbf{A}}\right) = \mathbf{X}_D^{-1}\frac{d\vec{\mathbf{X}}_D}{d\vec{\mathbf{A}}}\mathbf{A} = -\left(\mathbf{NVF}_D\right)^{-1}\mathbf{NV}$$

The sensitivity matrix of fluxes with respect to rate constants can be expressed as follows: The total derivative of the flux vector with respect to rate constant vector is

$$\frac{d\vec{\mathbf{V}}}{d\vec{\mathbf{A}}} = \frac{\partial\vec{\mathbf{V}}}{\partial\vec{\mathbf{X}}_D}\frac{d\vec{\mathbf{X}}_D}{d\vec{\mathbf{A}}} + \frac{\partial\vec{\mathbf{V}}}{\partial\vec{\mathbf{A}}}$$

Normalization yields

$$\mathbf{V}^{-1}\frac{d\vec{\mathbf{V}}}{d\vec{\mathbf{A}}}\mathbf{A} = \mathbf{V}^{-1}\frac{\partial\vec{\mathbf{V}}}{\partial\vec{\mathbf{X}}_D}\mathbf{X}_D\mathbf{X}_D^{-1}\frac{d\vec{\mathbf{X}}_D}{d\vec{\mathbf{A}}}\mathbf{A} + \mathbf{V}^{-1}\frac{\partial\vec{\mathbf{V}}}{\partial\vec{\mathbf{A}}}\mathbf{A}$$

Flux sensitivity matrix with respect to rate constants is therefore obtained as

$$\mathbf{S}\left(\vec{\mathbf{V}},\vec{\mathbf{A}}\right) = \mathbf{V}^{-1}\frac{d\vec{\mathbf{V}}}{d\vec{\mathbf{A}}}\mathbf{A} = \mathbf{F}_D\,\mathbf{S}\left(\vec{\mathbf{X}}_D,\vec{\mathbf{A}}\right) + \mathbf{I}$$

The flux connectivity relationships are

$$\begin{aligned}\mathbf{S}\left(\vec{\mathbf{V}},\vec{\mathbf{A}}\right)\mathbf{F}_D &= \left(\mathbf{F}_D\,\mathbf{S}\left(\vec{\mathbf{X}}_D,\vec{\mathbf{A}}\right) + \mathbf{I}\right)\mathbf{F}_D \\ &= \left(-\mathbf{F}_D\left(\mathbf{NVF}_D\right)^{-1}\mathbf{NV} + \mathbf{I}\right)\mathbf{F}_D \\ &= -\mathbf{F}_D\left(\mathbf{NVF}_D\right)^{-1}\mathbf{NVF}_D + \mathbf{F}_D = \mathbf{0}\end{aligned}$$

The proof is complete. Further details of these derivations are discussed in Voit (2000) and Cascante, et al. (1991).

**References**


1. Cascante, M., Franco, R. and Canela, E. (1991) Sensitivity analysis: a common foindation of theories for the quantitative study of metabolic control, in Voit, E.O. (Ed.), Canonical nonlinear modeling. Van Nostrand Reinhold, New York, Ch.4.
2. Voit, E.O. (2000) Computational Analysis of Biochemical Systems, Cambridge University Press, Chapter 7.
