# Supplementary material for: Estimating parameters for generalized mass action models with connectivity information
Source: BMC Bioinformatics. 2009 May 11;10:140. doi: 10.1186/1471-2105-10-140 (PMC2694188; doi:10.1186/1471-2105-10-140)
Supplement: Additional file 2 — Hybrid differential evolution. To describes the computational algorithm and the property of convergence for the HDE algorithm. [file 1471-2105-10-140-S2.pdf]

Supplement 2: Hybrid differential evolution

Estimating Parameters for Generalized Mass Action Models with Connectivity Information

Chih-Lung Ko, Eberhard O. Voit and Feng-Sheng Wang

Hybrid differential evolution (HDE) is illustrated in the following, which describes the computational algorithm and the property of convergence for the algorithm.

Algorithm

A nonlinear optimization problem is formulated as

$$\min_{\boldsymbol{\theta}} P(\boldsymbol{\theta}) \qquad \text{(S2-1)}$$

where $\boldsymbol{\theta} \in \Omega$ is a vector of $n \times 1$ decision parameters. The objective function $P(\boldsymbol{\theta})$ is defined on the search space $\Omega \subseteq \Re^n$, which is defined as an *n*-dimensional rectangle in $\Re^n$

$$\boldsymbol{\theta}_{\min} \leq \boldsymbol{\theta} \leq \boldsymbol{\theta}_{\max} \qquad \text{(S2-2)}$$

where $\boldsymbol{\theta}_{\min}$ and $\boldsymbol{\theta}_{\max}$ are the lower and upper bounds of the decision parameters, respectively.

Hybrid differential evolution (HDE) is quite a simple population based, stochastic function method that constitutes an extension from the original algorithm of DE as introduced by Storn and Price (1996; 1997). The original algorithm of DE is used to solve the unconstrained nonlinear optimization problems. Chiou and Wang (1997; 1999) have extended the original DE to solve optimal control problems. The basic operations of DE are similar to conventional evolutionary algorithms (EAs), as listed in Table S2-1.

Table S2-1. Basic operations for differential evolution and hybrid differential evolution

Differential Evolution	Hybrid Differential Evolution
1. Representation and initialization<br>2. Mutation<br>3. Crossover<br>4. Selection and evaluation<br>5. Repeat steps 2 to 4	1. Representation and initialization<br>2. Mutation<br>3. Crossover<br>4. Restriction<br>5. Selection and evaluation<br>6. Acceleration if necessary<br>7. Migration naturally or enforced if necessary<br>8. Repeat steps 2 to 6

For an evolutionary computation, two factors are important in the evolution process: population diversity and selective pressure. As population diversity is increased (i.e., a larger population is used), the genotype of the offspring differs more in comparison to that of the parent. Therefore, high population diversity can increase the opportunity to explore the global optimum and avoids premature convergence to the local optimum. As selective pressure is increased (i.e., use a smaller population), the search focuses only on a small population. Hence, highly selective pressure can decrease the computational overhead and increase the chance to rapidly approach a (possibly local) optimum. To balance these two important factors (i.e., simultaneously increase the solution quality and convergence rate), a widespread search heuristic (called migration or diversification) and a local search heuristic

(called acceleration) are embedded in HDE to facilitate a global search. The basic operations of HDE are also listed in Table S2-1. Each operation is illustrated as follows:

1. Representation and Initialization

The HDE structure is a parallel direct search algorithm which utilizes N_p vectors of the decision parameters $\boldsymbol{\theta}$ in the optimization problem, i.e., $\boldsymbol{\theta}_i^G, i = 1,.2,..., N_p$, as a population for each generation *G*. The initial population is randomly selected and should attempt to cover the entire search space uniformly as shown in the following form:

$$\boldsymbol{\theta}_i^0 = \boldsymbol{\theta}_{\min} + \rho_i(\boldsymbol{\theta}_{\max} - \boldsymbol{\theta}_{\min}), i = 1,..., N_p \tag{S2-3}$$

where ρ_i denotes the uniformly distributed random numbers.

2. Mutation

The mutation operation of DE and HDE is the essential component, compared with the other evolutionary algorithms. The mutation process in DE and HDE uses the difference between two or four randomly selected individuals to create a search direction. The mutation process at the (G-1)th generation begins by randomly selecting either two or four mutually independent individuals, $\boldsymbol{\theta}_j$, $\boldsymbol{\theta}_k$, $\boldsymbol{\theta}_l$, and $\boldsymbol{\theta}_m$. Each selected individual pair, $\boldsymbol{\theta}_j$ and $\boldsymbol{\theta}_k$, at the (G-1)th generation is employed to define a difference vector $\mathbf{D}_{jk}$ as

$$\mathbf{D}_{jk} = \boldsymbol{\theta}_j^{G\text{-}1} - \boldsymbol{\theta}_k^{G\text{-}1} = \begin{bmatrix} \theta_1 \\ \vdots \\ \theta_{n_\theta} \end{bmatrix}_j^{G-1} - \begin{bmatrix} \theta_1 \\ \vdots \\ \theta_{n_\theta} \end{bmatrix}_k^{G-1} \tag{S2-4}$$

Four mutually independent individuals are then combined to form a difference vector $\mathbf{D}_{jklm}$ as

$$\mathbf{D}_{jklm} = \mathbf{D}_{jk} + \mathbf{D}_{lm} = \left(\boldsymbol{\theta}_j^{G\text{-}1} - \boldsymbol{\theta}_k^{G\text{-}1}\right) + \left(\boldsymbol{\theta}_l^{G\text{-}1} - \boldsymbol{\theta}_m^{G\text{-}1}\right) \tag{S2-5}$$

A perturbed or mutant individual $\hat{\boldsymbol{\theta}}_i^{G\text{-}1}$ is therefore generated based on the parent individual $\boldsymbol{\theta}_p^{G\text{-}1}$ in the mutation process by

$$\hat{\boldsymbol{\theta}}_i^{G\text{-}1} = \boldsymbol{\theta}_p^{G\text{-}1} + F\,\mathbf{D}_{jklm}, i = 1,..., N_p \tag{S2-6}$$

Here, we refer to the mutation process (S2-6) by the difference of the selected individuals as the differential mutation strategy.

In DE, a differential mutation factor $F \in (0, 1.2]$ is fixed and should be set by the user to ensure the fastest possible convergence. However, the mutation factor in HDE is randomly selected at every generation to obtain a more perturbed individual. Figure S2-1 shows a two-dimensional example that illustrates the mutually different individuals that play a part in the generation of the mutant individual. The difference of two random individuals acts as a search direction in the solving space. The mutation factor selected between zero and one is used to yield a perturbation to ensure the fastest possible convergence. As a result, the mutant individual in (S2-6) is essentially a perturbed replica of the parent individual. Table S2-2 lists five common used differential mutation strategies in DE and HDE. The sixth mutation strategy was introduced by Liao *et al.*, (2001) to solve mixed-integer optimization problems. A linear crossover for the ith individual and the best individual is first applied to yield the parent individual, and then the parent individual is employed to generate the next mutant individual. This mutation strategy has been succeeded to solve several mixed-integer and real-valued optimization problems (Liao *et al.*, 2001; Wang et al., 2001; Chen and Wang, 2003; Lin and Wang, 2007; Cheng and Wang, 2008).

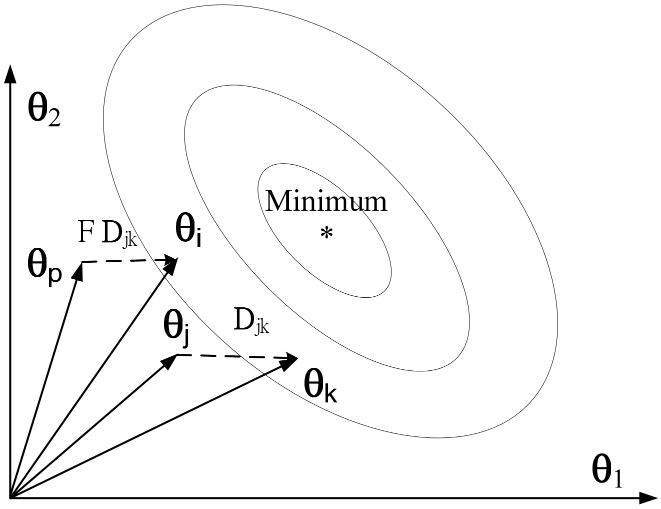

Figure S2-1. An example of a two-dimensional objective function showing its contour and the operation for generating mutant vectors $\boldsymbol{\theta}_i$. $\mathbf{D}_{jk}$ is the direction of differential variation. F $\mathbf{D}_{jk}$ is a perturbation.

Table S2-2. Various differential mutation strategies in differential evolution and hybrid differential evolution. The linear combination factor ρ in the sixth strategy is a random number between [0, 1].

Item	Mutation strategy
1	$\hat{\boldsymbol{\theta}}_i^{G\text{-}1} = \boldsymbol{\theta}_{best}^{G-1} + F(\boldsymbol{\theta}_j^{G\text{-}1} - \boldsymbol{\theta}_k^{G\text{-}1})$
2	$\hat{\boldsymbol{\theta}}_i^{G\text{-}1} = \boldsymbol{\theta}_i^{G\text{-}1} + F(\boldsymbol{\theta}_j^{G\text{-}1} - \boldsymbol{\theta}_k^{G\text{-}1})$
3	$\hat{\boldsymbol{\theta}}_i^{G\text{-}1} = \boldsymbol{\theta}_i^{G\text{-}1} + F[(\boldsymbol{\theta}_{best}^{G-1} - \boldsymbol{\theta}_i^{G\text{-}1}) + (\boldsymbol{\theta}_j^{G-1} - \boldsymbol{\theta}_k^{G\text{-}1})]$
4	$\hat{\boldsymbol{\theta}}_i^{G\text{-}1} = \boldsymbol{\theta}_{best}^{G-1} + F[(\boldsymbol{\theta}_j^{G\text{-}1} - \boldsymbol{\theta}_k^{G\text{-}1}) + (\boldsymbol{\theta}_l^{G\text{-}1} - \boldsymbol{\theta}_m^{G\text{-}1})]$
5	$\hat{\boldsymbol{\theta}}_i^{G\text{-}1} = \boldsymbol{\theta}_i^{G\text{-}1} + F[(\boldsymbol{\theta}_j^{G\text{-}1} - \boldsymbol{\theta}_k^{G\text{-}1}) + (\boldsymbol{\theta}_l^{G\text{-}1} - \boldsymbol{\theta}_m^{G\text{-}1})]$
6	$\hat{\boldsymbol{\theta}}_i^{G\text{-}1} = [\rho\boldsymbol{\theta}_{best}^{G\text{-}1} + (1-\rho)\boldsymbol{\theta}_i^{G\text{-}1}] + F(\boldsymbol{\theta}_j^{G\text{-}1} - \boldsymbol{\theta}_k^{G\text{-}1})$

3. Crossover

Based on the mutation operation in (S2-6), the mutant vector $\hat{\boldsymbol{\theta}}_l^{G-1}$ is a noisy replica of $\boldsymbol{\theta}_p^{G-1}$. However, due to inbreeding, the differential mutation is highly predisposed to a fast decrease of population diversity, which leads to the undesirable premature convergence. The binomial crossover operation in DE and HDE is employed to increase the local population diversity, which is similar to conventional evolutionary algorithms. The binomial crossover is expressed as

$$\theta_{ji}^{G} = \begin{cases} \theta_{ji}^{G-1}, & \text{if a random number } > C_R \\ \hat{\theta}_j^{G-1}, & \text{otherwise}, j = 1,...,n; i = 1,..., N_p \end{cases} \quad \text{(S2-7)}$$

where the crossover factor $C_R \in [0, 1]$ is set by the user. Figure S2-2 provides a pictorial representation of the crossover mechanism. This figure illustrates that the newly generated individual $\boldsymbol{\theta}_i^G$ not only retain original features but also locally increase gene diversity.

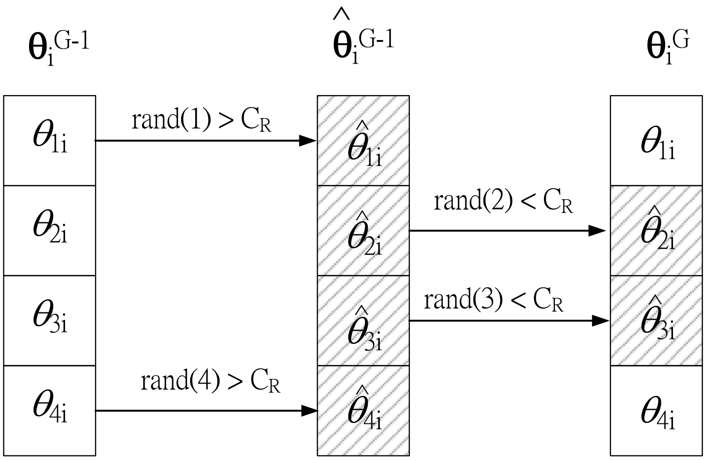

Figure S2-2. Illustration of the crossover operation for a crossover factor CR. rand(i) stands for a random number.

4. Restriction

The original code of the DE algorithm (Storn and Price, 1996; 1997) did not check if the newly generated individuals were within their bound regions; therefore, the restriction for each decision parameter was added into the HDE algorithm.

5. Selection and Evaluation

The evaluation operation in HDE and DE consists of two selection steps. The first selection step is one-to-one competition. The next step selects the best individual in the population. The first selection operation is a one-to-one competition between the parent and its offspring. This process of determining which trial individuals ought to survive in the next generation is straightforward. This competition means that any parent will be replaced by its offspring if its fitness is worse than that of its offspring. On the other hand, any parent will be retained in the next generation if its fitness is better than its offspring. Here, the better fitness means a smaller objective function value. Both selection steps are therefore expressed as

$$\boldsymbol{\theta}_i^G = \arg\min\left\{P\left(\boldsymbol{\theta}_i^{G-1}\right), P\left(\boldsymbol{\theta}_i^G\right)\right\}, i = 1, \ldots, N_p \tag{S2-8}$$

$$\hat{\boldsymbol{\theta}}_b^G = \arg\min\left\{P\left(\boldsymbol{\theta}_i^G\right), i = 1, \ldots, N_p\right\} \tag{S2-9}$$

6. Acceleration

Acceleration is used to speed up convergence. This operation is similar to a simulated annealing method to avoid a premature solution. Based on our experience using DE to solve optimization problems, the best fitness does not descend continuously from generation to generation. It usually descends toward a better fitness after several generations. In this situation, the acceleration can be used to speed up convergence. When the best fitness in the present generation is not improved any longer by the mutation and crossover operations, a descent method is applied to push the best individual toward obtaining a better solution.

Here, we use the steepest descent method with one-step search as a local search method in order to explain the accelerated operation. The operation is, therefore, expressed as

$$\boldsymbol{\theta}_b^G = \begin{cases} \hat{\boldsymbol{\theta}}_b^G, & \text{if } P\left(\hat{\boldsymbol{\theta}}_b^G\right) < P\left(\boldsymbol{\theta}_b^{G-1}\right) \\ \boldsymbol{\theta}_b^N = \hat{\boldsymbol{\theta}}_b^G - \alpha\, \nabla P, & \text{otherwise} \end{cases} \tag{S2-10}$$

where $\hat{\boldsymbol{\theta}}_b^G$ denotes the best individual, as obtained from equation (S2-9). The gradient of the objective function, ∇P, can be approximately calculated by finite difference. The step size $\alpha \in (0,1]$ in (S2-10) is determined by the descent property. Initially, α is set to a value of one to obtain the new individual $\boldsymbol{\theta}_b^N$. The objective function $P\left(\boldsymbol{\theta}_b^N\right)$ is then compared with $P\left(\boldsymbol{\theta}_b^G\right)$. If the descent property is followed, i.e.,

$$P\left(\boldsymbol{\theta}_b^N\right) < P\left(\boldsymbol{\theta}_b^G\right) \tag{S2-11}$$

then $\boldsymbol{\theta}_b^N$ becomes a candidate in the next generation and is added to this population replacing the worst individual. On the other hand, if the descent property fails, the step size is reduced by 0.5 or 0.8 and the descent method is repeated to obtain $\boldsymbol{\theta}_b^N$ until $\alpha \nabla P$ becomes sufficiently small or an iteration limit is exceeded. Consequently, according to equation (S2-10), the best fitness should be at least equal or smaller than $P\left(\boldsymbol{\theta}_b^{G-1}\right)$. Note that the steepest descent method is performed only for one-step descending to determine a better solution and is not applied to obtain a local minimum. If the user considers the stability and reliability of the numerical gradient using finite differences, an alternative direct local search method, such as the Hooke-Jeeves search method or simplex search method, can be used to ensure a better local tuning.

7. Migration

The rate of convergence can be improved by the acceleration. However, faster descent usually results in yielding a premature solution. In addition, performing this operation tends to gradually cluster the candidate individuals around the best individual so that the population diversity decreases. Furthermore, the closely clustered individuals cannot reproduce better individuals by mutation and crossover operations as observed from equations (S2-6) and (S2-7). The mutation operation in HDE is employed to the weighted difference between two random individuals to obtain the mutant individual. This differential mutation descends quickly so that it will make most individuals gradually cluster around the best candidate individual in some generations. As a consequence, the population diversity and the exploration capability diminish, and thus the clustered individuals are unable to reproduce more diversified individuals through the mutation operation because the weighted difference becomes nearly zero as observed from equation (S2-5). Furthermore, the recombination of the mutant individuals and their clustered parent is still unable to reproduce a diversified population through the crossover operation because the genes of the clustered individuals are similar as observed from (S2-7). In order to enhance the exploration capability of the search space and improve the selection pressure from the small population, the migration operation, a widespread search heuristic, is devised to generate a diversified population. The new candidates are regenerated on the basis of the best individual $\boldsymbol{\theta}_b^G$ as follows:

$$\boldsymbol{\theta}_i^G = \boldsymbol{\theta}_b^G + \mathbf{N}(0,\sigma),\ i = 1,\ldots,N_p;\ i \neq b \tag{S2-12}$$

where $\mathbf{N}(0,\sigma)$ denotes a vector of independent random Gaussian numbers with a mean of zero and standard deviation σ. Other types of regeneration which use non-uniform random numbers can be used. The jth gene of the ith individual is therefore regenerated

$$\theta_{ji} = \begin{cases} \theta_{jb} + \delta_{ji}(\theta_{j\min} - \theta_{jb}), & \text{if } \tilde{\delta}_{ji} < \dfrac{\theta_{jb} - \theta_{j\min}}{\theta_{j\max} - \theta_{j\min}} \\ x_{jb} + \delta_{ji}(\theta_{j\max} - \theta_{jb}), & \text{otherwise};\ j = 1,\ldots,n;\ i = 1,\ldots,N_p \end{cases} \tag{S2-13}$$

where δ_{ji} and $\tilde{\delta}_{ji}$ denote the uniformly distributed random numbers for the jth gene of the ith individual. Herein, $\theta_{j\min}$ and $\theta_{j\max}$ are expressed as the lower and upper bounds, respectively, of the jth gene of the decision parameters. This diversified population is then used as the initial decision parameters to escape the local optimum points.

The migration operation is performed only if a measure of population diversity fails to satisfy the desired tolerance; i.e., when most of the individuals cluster together, the migration must be actuated to make some improvements. In HDE, we propose a measure called the degree of population diversity to check whether the migration operation should be performed. In order to define the degree of population diversity, we first introduce the following gene diversity index to distinguish the similarity between the best individual and the other individuals. The gene diversity index is defined as

$$\eta_{ji} = \begin{cases} 0, & \text{if } \left| \dfrac{\theta_{ji}^G - \theta_{jb}^G}{\theta_{jb}^G} \right| < \varepsilon_2;\ j = 1,\dots,n; i = 1,\dots,N_p \\ 1, & \text{otherwise} \end{cases} \qquad \text{(S2-14)}$$

where η_{ji} is the gene diversity index to describe similarity for the jth gene of the ith individual to the best individual, $\varepsilon_2 \in [0,1]$ is the tolerance provided by the user. Here, θ_{jb}^G is the jth gene of the best individual at the Gth generation. According to Eq.(S2-14), we set the gene diversity index for the jth gene of the ith individual to zero if this gene clusters around the best gene. We now define the degree of population diversity η as the ratio of the total diversified genes to the overall genes except for the best individual. From (S2-14), we have the degree of population diversity as

$$\eta = \left\{ \sum_{\substack{i=1 \\ i \neq b}}^{N_p} \sum_{j=1}^{n} \eta_{ji} \right\} / (n(N_p - 1)) \qquad \text{(S2-15)}$$

From equations (S2-14) and (S2-15), we observe that the degree of population diversity is between zero and one. A zero value implies that all genes are clustered around the best individual. A value of one indicates that the current candidate individuals form a completely diversified population. The desired tolerance for population diversity is assigned within this region. A tolerance value of zero implies that the migration operation is switched off. A tolerance value of one implies that the migration operation is performed in every generation. The user can set a tolerance value for population diversity, $\varepsilon_1 \in [0,1]$, to assess whether the migration operation should be performed. If η is smaller than ε_1, then HDE performs the migration operation to generate a new population so as to escape the premature trap. If η is not less than ε_1, then HDE suspends the migration operation to maintain a constant search direction toward a target.

Figure S2-3 shows a numerical example to describe the concept of degree of population diversity. Suppose we attempt to minimize a function with two-dimensional parameters $\boldsymbol{\theta} = [\theta_1, \theta_2]$. Suppose further that the tolerance, ε_2, of the gene diversity is set to 0.1. Assume that after some generations of HDE, we obtain the following population

$$\boldsymbol{\theta}_1 = [1.0 \quad 1.0],\ \boldsymbol{\theta}_2 = [1.05 \quad 3.0],\ \boldsymbol{\theta}_3 = [1.3 \quad 2.0],\ \boldsymbol{\theta}_4 = [1.5 \quad 1.06].$$

Suppose that the first individual $\boldsymbol{\theta}_1$ is the fittest. The degree of population diversity in this example is explained by the fact that two thirds of the genes in this population are dissimilar to the best individual. As presented, fewer dissimilar individuals make it harder to explore better offspring by the mutation and crossover operations. The fact results from a premature

convergence. Therefore, if the degree of population diversity is smaller than the assigned tolerance, the migration should be performed to regenerate the next diversified population.

The above-mentioned migration is performed only if the individuals are closely clustered. This operation refers to a natural migration. The algorithm may encounter a special situation in which all of the individuals are simultaneously trapped in a completely flat region of the solution space. This implies that η is not less than ε_1 but the finesses for all individuals are nearly identical, i.e., $P(\boldsymbol{\theta}_1) \cong P(\boldsymbol{\theta}_2) \cong \ldots \cong P(\boldsymbol{\theta}_{N_p})$. Hence, natural migration cannot be actuated and the algorithm stays locked in this region indefinitely. At this time, an enforced migration similar to (S2-12) or (S2-13) must be performed to escape this flat region.

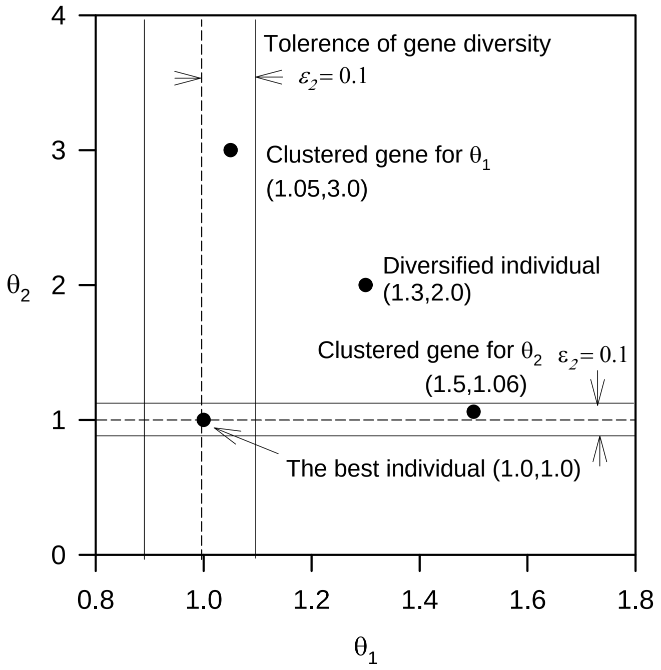

Figure S2-3. Illustration of the gather gene and diversified individual with respect to the best individual.

Property of convergence

The convergence of evolutionary algorithms is one of the most challenging theoretical issues in the evolutionary computation area, and several researchers have explored this problem from different perspectives (Michalewicz, 1996; Schwefel, 1994). Under some assumptions, HDE can be shown as a method using N_p parallel processors of the two member evolution strategy ((1+1)-ES). From this standpoint, the global convergence of HDE can be easily inspected.

The following assumptions are considered for HDE. The migration phase is performed at every generation; i.e., the tolerance for the population diversity is set to one. In addition, the regenerated population is generated with a Gaussian random number with mean 0 and standard deviation $\sigma / \gamma F$. The best individual is used in the mutation operation. The crossover operation in HDE is turned off, *i.e.*, $C_R = 1$. Under the above assumptions, the proposed operations become an algorithm of N_p parallel processors of (1+1)-ES with one step descent operation. This algorithm is summarized in Table S2-3.

Substituting the migration operation step in Table S2-2 into the mutation operation in Table S2-3, we obtain N_p parallel processors of (1+1)-ES as:

$$\boldsymbol{\theta}_i^G = \boldsymbol{\theta}_i^{G-1} + \mathbf{N}(0,\sigma),\ i=1,\ldots,N_p \tag{S2-16}$$

If one processor is used, *i.e.*, $N_p = 1$, this algorithm becomes the classical (1+1)-ES as discussed in Schwefel (1994). The global convergence property for the classical (1+1)-ES has been shown by Schwefel (1994). Since this special algorithm of HDE is composed of N_p parallel processors of (1+1)-ES, such convergence property can be cited for each processor. This implies that the global minimum can be achieved with sufficiently long search generations for each processor.

Table S2-3. N_p parallel processor for two member evolution strategy.

step	Procedure
1	initialization.<br>$\boldsymbol{\theta}_i^0 = \boldsymbol{\theta}_{\min} + \rho_i(\boldsymbol{\theta}_{\max} - \boldsymbol{\theta}_{\min}),\ i=1,\ldots,N_p$
2	migration operation.<br>$\boldsymbol{\theta}_i^{G-1} = \boldsymbol{\theta}_b^{G-1} + \mathbf{N}(0,\sigma/\gamma F),\ i=1,\ldots,N_p;\ i \ne b$
3	mutation operation.<br>$\boldsymbol{\theta}_i^G = \boldsymbol{\theta}_i^{G-1} + F(\boldsymbol{\theta}_b^{G-1} - \boldsymbol{\theta}_i^{G-1} + \boldsymbol{\theta}_k^{G-1} - \boldsymbol{\theta}_l^{G-1}),\ i=1,\ldots,N_p;\ i \ne k,l$
4	evaluation operation.<br>$\boldsymbol{\theta}_i^G = \arg\min\left\{P\left(\boldsymbol{\theta}_i^{G-1}\right), P\left(\boldsymbol{\theta}_i^G\right)\right\},\ i=1,\ldots,N_p$<br>$\hat{\boldsymbol{\theta}}_b^G = \arg\min\left\{P\left(\boldsymbol{\theta}_i^G\right), i=1,\ldots,N_p\right\}$
5	acceleration operation.<br>$\boldsymbol{\theta}_b^G = \begin{cases} \hat{\boldsymbol{\theta}}_b^G, & \text{if } P\left(\hat{\boldsymbol{\theta}}_b^G\right) < P\left(\boldsymbol{\theta}_b^{G-1}\right) \\ \boldsymbol{\theta}_b^N = \hat{\boldsymbol{\theta}}_b^G - \alpha\,\nabla P, & \text{otherwise} \end{cases}$
6	repeat of steps 2 to 5.

In the acceleration operation, the best individual is used to find the next best individual by a descent method. A convergent sequence can be generated by the descent mapping as observed from equation (S2-10). According to the constrictive mappings in (S2-10) and (S2-16), the global convergence for this special algorithm of HDE is completely proved by using the theorem of composition of mappings (Luenberger, 1984).

References

1. Chen, Y. F. and Wang, F. S. (2003). Crisp and fuzzy optimization of a fed-batch fermentation for ethanol production. *Industrial & Engineering Chemistry Research*, **42**, 6843-6850.
2. Cheng H.C. and Wang, F.S. (2008). Optimal biocompatible solvent design for a two-stage extractive fermentation process with cell recycling, *Computers & Chemical Engineering*, **32**, 1385-1396.
3. Chiou, J.P. and Wang, F.S. (1997). Hybrid differential evolution for parameter estimation of a batch bioprocess, *IEEE Singapore International Symposium on Control Theory and Applications,* Singapore, 171-174.
4. Chiou, J. P. & Wang, F. S. (1999). Hybrid method of evolutionary algorithms for static and dynamic optimization problems with application to a fed-batch fermentation process. *Computers & Chemical Engineering*. **23**, 1277-1291.
5. Liao, C. T., Tzeng, W. J., & Wang, F. S. (2001) Mixed-integer hybrid differential evolution for synthesis of chemical processes. *Journal of the Chinese Institute of Chemical Engineers*, **32**, 491–502.
6. Lin, H.T. & Wang, F.S. (2007). Optimal design of an integrated fermentation process for lactic acid production, *AIChE Journal*, **53**, 449–459.
7. Luenberger, D.G. Linear and nonlinear programming, Addison-Wesley, New York (1984).
8. Michalewicz, Z. Genetic algorithms + data structures = evolution programs, Springer-Verlag, New York (1996).
9. Schwefel, H.P. Evolution and optimum seeking. John Wiley, New York (1994).
10. Storn, R. and Price, K.V. (1996). Minimizing the real functions of the ICEC '96 contest by differential evolution. *IEEE Conf. on Evolutionary Computation,* Nagoya, 842-844.
11. Storn, R. and Price, K. V. (1997). Differential evolution: a simple and efficient heuristic for global optimization over continuous spaces, *Journal Global Optimization*, **11**, 34 1-369.
12. Wang, F. S., Su, T. L. and Jang, H. J., 2001, Hybrid differential evolution for problems of kinetic parameter estimation and dynamic optimization of an ethanol fermentation process, *Industrial and Engineering Chemistry Research,* **40,** 2876-2885.
